# Changes of Adult Population Health Status in China from 2003 to 2008

**DOI:** 10.1371/journal.pone.0028411

**Published:** 2011-12-02

**Authors:** Hongpeng Sun, Qiuju Zhang, Xiao Luo, Hude Quan, Feng Zhang, Chang Liu, Meina Liu

**Affiliations:** 1 Department of Biostatistics, School of Public Health, Harbin Medical University, Harbin, People's Republic of China; 2 Department of Community Health Sciences and Centre for Health and Policy Studies, University of Calgary, Calgary, Canada; Fundación para la Prevención y el Control de las Enfermedades Crónicas No Transmisibles en América Latina (FunPRECAL), Argentina

## Abstract

**Objectives:**

The purpose of this study was to examine the change in health status of China's adult population between the years of 2003 and 2008 due to rapid economic growth and medical system improvement.

**Methods:**

Data from the third and fourth Chinese national health services surveys covering 141,927 residents in 2003 and 136,371 residents in 2008 who were aged >18 years were analyzed.

**Results:**

Chinese respondents in 2008 were more likely to report disease than in 2003. Smoking slightly decreased among men and women, and regular exercise showed much improvement. Stratified analyses revealed significant subpopulation disparities in rate ratios for 2008/2003 in the presence of chronic disease, with greater increases among women, elderly, the Han nationality, unmarried and widow, illiterate, rural, and regions east of China than other groups.

**Conclusions:**

Chinese adults in 2008 had worse health status than in 2003 in terms of presence of chronic disease. China's reform of health care will face more complex challenges in coming years from the deteriorating health status in Chinese adults.

## Introduction

In recent years, the healthcare system in China has significantly improved [1], [2], [3], [4]. In 2001, Beijing succeeded in the bid to host the 2008 Olympic Games. Subsequently, Chinese government and civil organizations launched extensive fitness programs for the general public to raise the level of physical activity and health. As a result, more sports facilities were built with free access in the community, there was increased parkland, and news media also participated in this campaign [5]. China suffered greatly in the 2003 when the worldwide prevalence of Severe Acute Respiratory Syndrome (SARS) took a sharp rise. Since that lesson, there is evidence that Chinese people have improved their general and personal hygiene and diet, undertaken more intensive physical exercise, and increased the overall frequency of hand washing [6]. Over the next 5 years, following 2003, Chinese healthcare systems have made significant reforms, and reflected on the vulnerability of public health [7], [8].

Available health resources in China have been continuously increasing along with China's dramatic economic development [9], [10], [11]. From 2003 to 2009, the medical insurance system in China underwent rapid development and the government input among total health expenditure increased by 17%. A new type of rural cooperative medical care system, a form of community-based health insurance for the rural population, was piloted in some counties in 2003; coverage rate reached 72.6% by December 2004, and would gradually cover all rural residents by 2010 [12], [13]. At the same time, urban resident health insurance scheme included 80% of cities by the end of 2007. Urban employee basic health insurance scheme in China has covered all cities since 2008 [13], [14], [15]. With healthcare reform deepening, these systems made the proportion of out-of-pocket payments drop to 30% and will be further reduced in future [13].

During the last decade, the health status of China's population has been changing. Chinese life expectancy increased from 71.4 years in 2000 to 73 years in 2005; infant mortality decreased from 25.5/1000 live births in 2003 to 15.3/1000 live births in 2007 while maternal mortality declined from 51.3/100,000 to 36.3/100,000 live births at the same time [16]. Although infectious disease, malnutrition, child, and maternal mortality rates have decreased, chronic and noncommunicable disease mortality has gradually increased [17]. For example, cancer and stroke are the major causes of death, accounting for 44.8% of all deaths in 2008, followed by respiratory disease and heart disease; chronic and noncommunicable disease mortality increased from 76.5% of all-cause death in 2003 to 82.5% in 2008 [18].

On the other hand, such indicators are insensitive to nonfatal conditions that contribute indirectly to death. Whether Chinese overall health status changed during the period from 2003 to 2008 remains unresolved. Hence it is imperative to assess population health using indicators that reflect contemporary health issues. This study aimed to describe the male and female adult Chinese population health status in multiple dimensions, including overall morbidity, presence of illness in the last 2 weeks and chronic disease in the last 6 months, and healthy behavior as regards smoking, alcohol consumption, and physical activity, using data from the most recent National Health Services Surveys by the Chinese government in 2003 and 2008. The main objective of these surveys was to measure performance of health systems and forecast health demand and long-term health problems. Our study provided important information for furthering research questions for future studies.

## Methods

Data deriving from the China Third National Health Services Survey in 2003 and the China Fourth National Health Services Survey in 2008 were assessed. The Chinese National Health Services Survey, the largest statewide health survey in China, collects data on multiple public health issues including health status, health behaviors, access to healthcare, and healthcare utilization. The survey respondent age and sex composition was comparable with the 2000 census. We included men and women aged >18 years and excluded those with missing values, resulting in a total of 141,927 (M/F, 70,541/71,386) residents in 2003 and 136,371 (66,801/69,570) in 2008.

### Data

The China National survey employed a multistage cluster sampling to select the sample randomly. The mainland of China was clustered according to the government administrative geographic system [19]. Both surveys were conducted in the same sampling areas, whereas all households were randomly selected again. First, 94 counties and cities were randomly selected from rural and urban areas (95 areas were selected in 2003). Second, 5 towns were selected from each county and 5 communities from each city respectively, resulting in 470 towns or communities. Third, 2 villages in each town and 2 neighborhoods in each community were randomly selected. Fourth, 60 households were randomly selected in each village or neighborhood, resulting in about 56,400 households in 2008 (57,000 households in 2003). During the survey, candidate households that could not be contacted by interviewers on three calls on different days were replaced. Survey completion rate was maintained at >95%. Interviewers, who were trained, explained the purposes and confidentiality of the survey then invited family members to participate. Residents could choose not to participate and their participation in the survey was accepted as oral consent. Adult residents were themselves required to answer questions; if they were not at home at the time of the survey then their nearest relative could serve as proxy. As a result, the survey response rate for adults was 83% in 2008 (77.8% in 2003).

Completeness of questionnaires was checked by a district survey manager at the end of each day. If there was missing information and errors on the survey, probands could be re-surveyed the next day. A 5% sample of households was randomly selected and re-surveyed to examine survey quality by telephone or visit again; the agreement was 95%.

The quality of survey data and consistency check demonstrated a Myer's index of 3.48; there was no age preference in the survey. The results of goodness-of-fit showed that the sample was not significantly different with the general population in age distribution. Similarity coefficient and GINI concentration ratio indicated that family size in the survey was consistent with the established national picture.

### Measures

Demographic variables included sex, age, ethnicity, marital status, education level, rural/urban residency, and geographic region. Ethnicity was grouped into Han or minority. Educational level was categorized into five categories such as illiterate, elementary school, junior high school, senior high school, and college or university or higher. China was geographically grouped into urban (city) or rural areas (town or village) after the governmental administration system, as well as eastern China, mid-China, and western China based on economic development status; eastern China is considered the most developed region, mid-China less developed, and western China the least.

Information about smoking, alcohol consumption, and physical exercise were collected. Presence of illness in the previous 2 weeks and physician-diagnosed chronic diseases in the last 6 months was recorded. The same survey method was used in both the 2003 and 2008 surveys. Definitions of variables have been described in detail elsewhere [20], [21].

### Statistical Analysis

All analyses were conducted separately for women and men, urban and rural, and total. Descriptive statistics were used to test the statistical differences in sociodemographic characteristics, smoking, alcohol consumption, physical exercise, presence of illness (in the previous 2 weeks) and chronic disease between the two surveys. Because of the large sample size and multiple categories in sociodemographic characteristics variables, P-values for differences between the two surveys were not reported. Finally, multiple binomial regressions with a log link were used to generate adjusted P-values to examine whether Chinese residents of 2008 had better health status compared with in 2003. Clustering of individuals within family was adjusted for using Generalized Estimating Equations in SAS 9.1 Proc GENMOD [22], [23]. Stratified analyses by sociodemographic variables were conducted for chronic disease to determine whether adjusted rate ratio (RR) and 95% confidence interval (95%CI) for 2008/2003 differed across strata.

## Results

### Demographic Characteristics

Demographic characteristics for residents are presented in Table 1. There was a difference in composition of some age groups between the two surveys: 18–34 age group (25.3% vs. 30.9%); 55–64 age group (15.9% vs. 12.1%); and ≥65 age group (14.8% vs. 12.9%). There were more elderly people in 2008 than 2003. More people in 2008 were divorce and widowed (1.5%, 7.5% vs. 1.1%, 6.6%). More people had higher education in 2008; the proportion of illiteracy was 20.0% in 2003 but 16.3% in 2008. On the other hand, male and female respondents, the composition of rural/urban, and region were similar between 2003 and 2008.

**Table 1:** Characteristics of survey respondents aged >18 years in China, 2003 and 2008.

	Men		Women		Total	
	2008 N (%)	2003 N (%)	2008 N (%)	2003 N (%)	2008 N (%)	2003 N (%)
N	66801	70541	69570	71386	136371	141927
**Age**						
18–34	16828 (25.2)	21786 (30.9)	17723 (25.5)	22004 (30.8)	34551 (25.3)	43790 (30.9)
35–44	15272 (22.9)	15809 (22.4)	16290 (23.4)	16417 (23.0)	31562 (23.1)	32226 (22.7)
45–54	14099 (21.1)	15220 (21.6)	14415 (20.7)	15210 (21.3)	28514 (20.9)	30430 (21.4)
55–64	10877 (16.3)	8838 (12.5)	10731 (15.4)	8345 (11.7)	21608 (15.9)	17183 (12.1)
≥65	9725 (14.5)	8888 (12.6)	10411 (15.0)	9410 (13.2)	20136 (14.8)	18298 (12.9)
**Minority**						
Han Chinese	57609 (86.2)	60835 (86.2)	59773 (85.9)	61390 (86.0)	117382 (86.1)	122225 (86.1)
Minority Chinese	9192 (13.3)	9706 (13.8)	9797 (14.1)	9996 (14.0)	18989 (13.9)	19702 (13.9)
**Marital status**						
Unmarried	9752 (14.6)	10495 (14.9)	6194 (8.9)	6538 (9.2)	15946 (11.7)	17033 (12.0)
Married	52876 (79.2)	56198 (79.7)	55276 (79.5)	57686 (80.8)	108152 (79.3)	113884 (80.2)
Divorce	1134 (1.7)	946 (1.3)	859 (1.2)	658 (0.9)	1993 (1.5)	1604 (1.1)
Widow	3039 (4.6)	2902 (4.1)	7241 (10.4)	6504 (9.1)	10280 (7.5)	9406 (6.6)
**Education**						
Illiterate	6359 (9.5)	8597 (12.2)	15869 (22.8)	19788 (27.7)	22228 (16.3)	28385 (20.0)
Elementary school	18567 (27.8)	19656 (27.9)	19112 (27.5)	19378 (27.2)	37679 (27.6)	39034 (27.5)
Junior high school	25484 (38.2)	26051 (36.9)	21426 (30.8)	20199 (28.3)	46910 (34.4)	46250 (32.6)
Senior high school	8807 (13.2)	8873 (12.6)	6848 (9.8)	6336 (8.9)	15655 (11.5)	15209 (10.7)
College or university	7584 (11.4)	7364 (10.4)	6315 (9.1)	5685 (8.0)	13899 (10.2)	13049 (9.2)
**Residence area**						
Urban	18673 (27.95)	19644 (27.85)	20323 (29.2)	20976 (29.4)	38996 (28.6)	40620 (28.6)
Rural	48128 (72.05)	50897 (72.15)	49247 (70.3)	50410 (70.6)	97375 (71.4)	101307 (71.4)
**Region of China**						
East of China	23104 (34.59)	24103 (34.17)	24443 (35.1)	24901 (34.9)	47547 (34.9)	49004 (34.5)
Middle of China	18891 (28.28)	19727 (27.97)	19617 (28.2)	19885 (27.9)	38508 (28.2)	39612 (27.9)
West of China	24806 (37.13)	26711 (37.87)	25510 (36.7)	26600 (37.3)	50316 (36.9)	53311 (37.6)

### Health Status

Presence of illness in the previous 2 weeks was 19.9% (M/F, 17.8%/21.9%; Table 2) in 2008, much higher than in 2003 (15.7%; M/F, 14.1%/17.4%). Both urban and rural had similar results. Similarly, presence of physician-diagnosed chronic disease in the last 6 months in 2008 was much higher than in 2003 (25.5% vs. 20.0%). Compared with 2003, the residents in 2008, by sex and residence area, had higher prevalence of diabetes, heart disease, stroke, and hypertension. Prevalence rate of cancer in rural area had a significant increase (0.2% vs. 0.1%). Male and urban residents in 2008 had less prevalence of peptic ulcer than 2003. Prevalence of rheumatoid arthritis in 2008 was higher in rural area compared with in 2003 (1.5% vs. 1.1%).

**Table 2:** Illness and morbidity in respondents aged >18 years in China, 2003 and 2008.

	Men		Women		Urban		Rural		Total	
	2008	2003	2008	2003	2008	2003	2008	2003	2008	2003
N	66801	70541	69570	71386	38996	40620	97375	101307	136371	141927
Presence of illness in the last 2 weeks before the survey*	11869 (17.8)	9960 (14.1)	15237 (21.9)	12383 (17.4)	8282 (21.2)	6438 (15.9)	18824 (19.3)	15905 (15.7)	27106 (19.9)	22343 (15.7)
Presence of physician diagnosed chronic disease in the last 6 months before the survey*	15321 (22.93)	12661 (18.0)	19388 (27.86)	15778 (22.1)	12966 (33.3)	11768 (29.0)	21743 (22.3)	16671 (16.5)	34709 (25.5)	28439 (20.0)
Infectious and parasitic disease	305 (0.5)	305 (0.4)	158 (0.2)	186 (0.3)	79 (0.2)	125 (0.3)	384 (0.4)	366 (0.4)	463 (0.3)	491 (0.3)
Cancer	184 (0.3)	125 (0.2)	164 (0.2)	113 (0.2)	151 (0.4)	121 (0.3)	197 (0.2)	117 (0.1)*	348 (0.3)	238 (0.2)*
Diabetes	866 (1.3)	476 (0.7) *	1017 (1.5)	598 (0.8) *	1263 (3.2)	476 (1.2) *	620 (0.6)	598 (0.6) *	1883 (1.4)	1074 (0.8) *
Heart disease	1193 (1.8)	1013 (1.4) *	1895 (2.7) *	1672 (2.3) *	1584 (4.1)	1613 (4.0) *	1504 (1.5)	1072 (1.1) *	3088 (2.3)	2685 (1.9) *
Stroke	899 (1.4)	702 (1.0) *	799 (1.2)	574 (0.8) *	627 (1.6)	702 (1.7)	1071 (1.1)	574 (0.6) *	1698 (1.3)	1276 (0.9) *
Chronic pulmonary disease	990 (1.5)	1174 (1.7) *	766 (1.1)	814 (1.1)	480 (1.2)	611 (1.5)*	1276 (1.3)	1377 (1.4)*	1756 (1.3)	1988 (1.4) *
Hypertension	4289 (6.4)	2215 (3.1) *	5369 (7.7)	2834 4.0) *	4646 (11.9)	2215 (5.5) *	5012 (5.2)	2834 (2.8) *	9658 (7.1)	5049 (3.6) *
Peptic ulcer	355 (0.5)	448 (0.6) *	226 (0.3)	266 (0.4)	129 (0.3)	170 (0.4)*	452 (0.5)	544 (0.5)	581 (0.4)	714 (0.5) *
Chronic liver disease	131 (0.2)	128 (0.2)	66 (0.1)	71 (0.1)	69 (0.2)	128 (0.3)	128 (0.1)	71 (0.1)	197 (0.1)	199 (0.1)
Chronic renal disease	106 (0.2)	85 (0.1)	165 (0.2)	177 (0.3)	96 (0.3)	85 (0.2)	175 (0.2)	177 (0.2)	271 (0.2)	262 (0.2)
Rheumatologic arthritis	570 (0.9)	540 (0.8)	1216 (1.8)	1084 (1.5)	332 (0.9)	540 (1.3)	1454 (1.5)	1084 (1.1) *	1786 (1.3)	1624 (1.1)

*Note: adjusted P value <0.001 for 2008 versus 2003 after adjustment for age, minority, marital status, education, gender (or residence area) and geographic region.

Compared with 2003, the proportion of current smokers in 2008 dropped from 27.7% to 26.3% (Table 3). However, the proportion of drank alcohol frequently was similar between 2008 and 2003. Chinese residents in 2008 were significantly more likely to perform regular exercise than in 2003 (14.6% vs. 11.3%). Compared with men, women were less likely to smoke and frequently drink alcohol in 2003 and 2008, and had more likelihood to perform regular exercise. Rural residents did less regular exercise than urban residents, and smoked more.

**Table 3:** Prevalence of smoking, alcohol intake, and physical activity in respondents aged >18 years in China.

	Men		Women		Urban		Rural		Total	
	2008	2003	2008	2003	2008	2003	2008	2003	2008	2003
N	66801	70541	69570	71386	38996	40620	97375	101307	136371	141927
Currently smoking*	33935 (50.8)	36900 (52.3)	1923 (2.8)	2431 (3.4)	9068 (23.3)	10144 (25.0)	26790 (27.5)	29187 (28.8)	35858 (26.3)	39331 (27.7)
Drank alcohol frequently (≥3 times per week)	11308 (16.9)	11580 (16.4)	845 (1.2)	809 (1.1)	2714 (7.0)	2993 (7.4)	9439 (9.7)	9396 (9.3)	12153 (8.9)	12389 (8.7)
Regular exercise in the last 6 months (≥3 times per week)*	9674 (14.5)	8151 (11.6)	10172 (14.6)	7828 (11.0)	14417 (37.0)	12517 (30.8)	5429 (5.6)	3462 (3.4)	19846 (14.6)	15979 (11.3)

*Note: adjusted P value <0.001 for 2008 versus 2003 after adjustment for age, minority, marital status, education, gender (or residence area) and geographic region.

### Stratified Analysis

After adjusting for independent variables listed in Table 1, residents in 2008 were still more likely to have chronic disease than in 2003 (risk-adjusted RR, 1.13; 95%CI, 1.11–1.15; Table 4). Stratified analyses showed significant subpopulation disparities in health status between 2003 and 2008. For male population presence of chronic disease rate ratios for 2008/2003 were higher among residents who were rural, 45–54 age group, Han Chinese, unmarried, illiterate, and in eastern China. For female population chronic disease rate ratios for 2008/2003 were higher among residents who were rural, aged >65 years, Han Chinese, unmarried, widowed, illiterate, and in eastern China.

**Table 4:** Adjusted RR and 95%CI for 2008 vs. 2003 in chronic disease in the last 6 months in different subpopulations*.

Subpopulation	Men	Women	Urban	Rural	Total
Total	1.12 (1.09, 1.14)	1.14 (1.12, 1.16)	1.05 (1.02, 1.08)	1.20 (1.17, 1.22)	1.13 (1.11, 1.15)
Sex					
Male	-	-	1.08 (1.04, 1.12)	1.15 (1.12, 1.19)	1.12 (1.09, 1.14)
Female	-	-	1.03 (1.00, 1.06)	1.23 (1.20, 1.27)	1.14 (1.12, 1.16)
Residence area					
Urban	1.08 (1.04, 1.12)	1.03 (1.00, 1.06)	-	-	1.05 (1.02, 1.08)
Rural	1.15 (1.12, 1.19)	1.23 (1.20, 1.27)	-	-	1.20 (1.17, 1.22)
Age					
18–34	0.97 (0.88, 1.08)	1.03 (0.93, 1.13)	0.88 (0.75, 1.04)	1.03 (0.95, 1.12)	1.00 (0.93, 1.08)
35–44	1.04 (0.96, 1.11)	1.01 (0.95, 1.07)	0.86 (0.78, 0.95)	1.08 (1.02, 1.14)	1.02 (0.97, 1.07)
45–54	1.19 (1.13, 1.25)	1.17 (1.12, 1.22)	1.06 (1.00, 1.13)	1.24 (1.19, 1.29)	1.18 (1.14, 1.22)
55–64	1.16 (1.11, 1.21)	1.15 (1.10, 1.19)	1.09 (1.03, 1.14)	1.20 (1.16, 1.26)	1.15 (1.12, 1.19)
≥65	1.11 (1.07, 1.14)	1.18 (1.14, 1.21)	1.07 (1.03, 1.10)	1.25 (1.20, 1.29)	1.14 (1.12, 1.17)
Minority					
Han	1.14 (1.12, 1.17)	1.17 (1.14, 1.19)	1.08 (1.06, 1.11)	1.22 (1.19, 1.25)	1.16 (1.14, 1.18)
Minority	0.91 (0.85, 0.98)	0.94 (0.89, 1.00)	0.58 (0.52, 0.65)	1.07 (1.01, 1.13)	0.93 (0.88, 0.97)
Marital status					
Unmarried	1.24 (1.08, 1.42)	1.17 (0.94, 1.45)	1.05 (0.90, 1.24)	1.16 (1.03, 1.31)	1.12 (1.01, 1.23)
Married	1.12 (1.09, 1.14)	1.12 (1.09, 1.14)	1.04 (1.01, 1.07)	1.18 (1.15, 1.21)	1.12 (1.10, 1.14)
Divorce	0.97 (0.81, 1.18)	0.91 (0.76, 1.09)	0.84 (0.71, 1.00)	1.11 (0.90, 1.38)	0.95 (0.83, 1.09)
Widow	1.11 (1.04, 1.19)	1.21 (1.17, 1.26)	1.12 (1.06, 1.17)	1.26 (1.20, 1.32)	1.19 (1.15, 1.23)
Education					
Illiterate	1.15 (1.09, 1.21)	1.22 (1.18, 1.25)	1.09 (1.03, 1.16)	1.23 (1.20, 1.27)	1.20 (1.17, 1.23)
Elementary school	1.14 (1.10, 1.19)	1.15 (1.11, 1.19)	1.05 (1.00, 1.10)	1.20 (1.16, 1.25)	1.15 (1.12, 1.18)
Junior high school	1.11 (1.06, 1.15)	1.08 (1.03, 1.13)	1.05 (1.00, 1.10)	1.15 (1.10, 1.21)	1.10 (1.06, 1.13)
Senior high school	1.07 (1.00, 1.15)	0.99 (0.92, 1.07)	1.02 (0.96, 1.09)	1.10 (0.99, 1.21)	1.04 (0.98, 1.09)
College or university	1.07 (1.01, 1.14)	0.98 (0.91, 1.05)	1.05 (0.99, 1.10)	0.93 (0.79, 1.10)	1.04 (0.99, 1.09)
Region of China					
East of China	1.20 (1.16, 1.24)	1.20 (1.17, 1.24)	1.18 (1.13, 1.22)	1.23 (1.18, 1.27)	1.20 (1.17, 1.23)
Middle of China	1.11 (1.07, 1.16)	1.16 (1.12, 1.21)	1.07 (1.03, 1.12)	1.19 (1.15, 1.24)	1.14 (1.10, 1.17)
West of China	1.04 (1.00, 1.08)	1.06 (1.03, 1.10)	0.81 (0.77, 0.85)	1.18 (1.14, 1.22)	1.05 (1.02, 1.08)

*Rate ratio for 2008 vs. 2003 adjusted for sex (or residence area), age, minority, marital status, education, and geographic region.

Rural residents had higher chronic disease rate ratios for 2008/2003 than urban residents (1.20 vs. 1.05), and the confidence intervals for the two rate ratios did not overlap; rate ratios for 2008/2003 were higher among rural residents who were >65 years, Han Chinese, widowed, illiterate, and in eastern China. Moreover, urban residents in eastern China also had higher RR (1.18; 95%CI, 1.13–1.22). However, presence of chronic disease in 2008 among residents in the 18–34 and 35–44 age groups, the divorced, and those in higher education was not significantly different compared with 2003. Notably, prevalence of chronic disease of minority nationality in 2008 was lower than in 2003, and especially in urban (RR, 0.58; 95%CI, 0.52–0.65).

## Discussion

Our review of data for the China third and fourth national health services surveys suggests conclusions in three broad areas. First, we found that Chinese population in 2008 was more likely to report illness compared with 2003, but less likely to smoke and more likely to do regular exercise. Second, chronic diseases in 2008 were more highly prevalent than in 2003, particularly hypertension, diabetes, heart disease, and stroke. Third, the disparity in the prevalence chronic disease of the two surveys was distinctly evident in different subpopulations.

In the current study, we found that prevalence rates of chronic diseases in the last 6 months before the surveys increased from 2003 to 2008. For example, the prevalence of hypertension and diabetes in 2008 approximately doubled since 2003, and that of stroke increased by 1.4 times. However, the prevalence of infectious disease did not change.

There are several possible explanations for the above findings. First, China's population has been in very rapid transition from a youthful to an aging population as life expectancy has increased [24], [25], [26]. Birth rates have fallen, and China's one-child family policy has been a strong driver of population aging [27]. The rapid decrease in China's birth rate, combined with stable or improving life expectancy, has led to an increasing proportion of elderly people. In 1982, only 7.6% of the population was aged >60 years; this proportion grew to 10.5% by 2000. In our study this proportion was 13.6% in 2003 and 15.4% in 2008. The UN predicts that >453 million Chinese will be aged >60 years by 2050 [28]. This so-called graying of China's population has increased the incidence rates of diseases associated with elderly populations, and will become more problematic in future [29].

Second, many of the known risk factors for chronic diseases have dramatically increased as societal change progresses. These behavioral elements include changing diets, levels of physical activity, and alcohol and tobacco consumption and have accelerated shift at a historically unprecedented pace and scale in China. Dietary grains intake decreased substantially, whereas that of meat, fat, and edible oil increased [30], [31], [32]. Consequently, obesity in Chinese people has increased as well as hypercholesterolemia [33], [34]. Obesity is a major public health problem since it contributes to development and exacerbation of major chronic diseases [35], [36], [37] including heart disease, type 2 diabetes, and some cancers. In addition, the increase in overweight people and obesity can be attributed to physical inactivity [38]. Physical activity helps a person maintain better posture and balance, stronger muscles and bones, more vitality, reduced stress, and continued independent living in later life [39]. Although the present research revealed that Chinese adults were more physically active in 2008 compared with 2003, only 14.6% of adult residents reported exercising ≥3 times per week. This may be due to improved access to physical activities or enhanced awareness of health. However, overall Chinese adult population health status has not been improved due to short time and small proportion of residents performing frequent exercise; hence it seems that the prevalence and burden of chronic diseases will continue to grow.

The third possible explanation is that the prevalence of hypertension in China has been rising rapidly during the period from 2003 to 2008. Our results indicate that the prevalence of hypertension in the last 6 months before the survey has doubled from 3.6% in 2003 to 7.1% in 2008; and 17.5% of Chinese people had been diagnosed with hypertension by medical doctors. However, the true prevalence of hypertension should be higher than reported because of respondents' unawareness of their condition. Previous national studies suggest that the prevalence of hypertension in the Chinese adult population has increased from 5.1% in 1959, to 7.7% in 1980, to 13.6% in 1991, and to 18.8% in 2002 [40], [41], [42]. Nevertheless they should be compared cautiously owing to methodological differences in sampling and to differences in the criteria used to define hypertension. In addition, control of hypertension in China is far from optimum. According to the national nutrition survey of 2002, 18.8% of Chinese adults had hypertension, and of the 24% affected individuals who were aware of their condition, 78% were treated and 19% adequately controlled [40], [42]. At the same time, 27.2% of diabetes patients took medication and 9.7% achieved controlled diabetes [43]. Therefore a national education program that can eliminate the huge gaps among presence, awareness, treatment, and ability to control of hypertension and diabetes should be given to the public, clinicians, and healthcare decision makers.

Fourth, insurance coverage that has been increasing in China, leading to higher health services utilization [13]. Previous national health services surveys revealed that the 2-week consultation rates were 11.8% of urban residents and 13.9% rural residents in 2003 [20]. The percentages increased to 12.7% of urban and 15.2% rural in 2008. Therefore Chinese adults in 2008 seemed more likely to detect disease.

The increasing range in the prevalence of chronic disease was more significant in women, Han Chinese, elderly, widowed, illiterate, rural, and eastern China than other subpopulations. The presence of chronic disease is rapidly increasing among more affluent people. For example rural residents, who have benefited from China's economic development similarly to urban populations, have experienced dramatic lifestyle changes in the past two decades. They are doing less physical work owing to mechanization and eating more high-protein or –fat food; and are therefore more likely to be aware of the presence of chronic disease and insurance coverage, especially the new rural cooperative medical care scheme, more health service utilization, and better transport have enabled rural residents to visit physicians [12], [13]. As a result, they are more likely to report illness.

Social determinants of health have become important factors associated with the decline of health status. Our findings suggest that to promote health status we should focus on elderly and widowed people and promote higher national educational level. Prevention strategies such as reduction of tobacco use, exposure to second-hand smoke, less dietary intake of salt and fat, and promotion of increased physical activity should also be prioritized.

There are four major limitations to this study. A major limitation is that data were collected through two cross-sectional surveys, whereas the two questionnaires did not have precisely the same structure. Also, the cross-sectional nature allows for errors in recall. A second limitation is that the present study was based on observational data; therefore we cannot be completely sure of the association between demographic characteristics and health status, even after controlling for potential confounders. A third limitation is that we only analyzed seven major risk factors but were unable to assess other important risk factors of diet and obesity. The fourth major limitation is that the prevalence of chronic disease was likely underestimated because only physician-diagnosed chronic disease in the last 6 months was recorded. However, this study mainly explored relative change for presence of chronic disease from 2003 to 2008. Some bias in both cross-sectional surveys could be offset to some extent.

Despite these limitations, our findings make a significant contribution to address the health characteristics of Chinese adult population. Additional research is needed to explore the reasons for the patterns we found, including how the outcomes examined in our study might differ for rural residents compared with urban residents. Future studies also might explore how types of health insurance might be related to health status.

In conclusion, our analysis demonstrates that Chinese adults in 2008 had worse health status than 2003 in terms of presence of various chronic diseases. Especially, prevalence of hypertension and diabetes in 2008 was twice that in 2003. However, smoking showed slight decrease among men and women, and regular exercise suggested much improvement. Our results also indicate that prevalence of chronic illnesses increased more among women, elderly, Han Chinese, unmarried and widowed, illiterate, rural, and those in eastern China. Public health policy should pay explicit attention to these issues. As economic development and environmental degradation continue, systems of disease prevention and health promotion in China will face ever-bigger difficulties and challenges.
